# MRI evaluation of ADC values and venous variations in fetal cerebral white matter with T2WI signal hyperintensity in late gestation

**DOI:** 10.3389/fmed.2026.1776880

**Published:** 2026-04-30

**Authors:** Yimin Cao, Jie Li, Juan Wang, Yi Xing, Haiqing Yang, Lixia Zhou

**Affiliations:** 1 Department of Medical Imaging, The Second Hospital of Hebei Medical University, Shijiazhuang City, China; 2 Medical School, Kunming University of Science and Technology, Kunming, China

**Keywords:** fetal magnetic resonance imaging (MRI), intrauterine distress, nervous system, neurodevelopmental disorders, prenatal diagnosis

## Abstract

**Background and purpose:**

We observed that some late-term fetal prenatal magnetic resonance imaging (MRI) scans revealed normal brain structures but exhibited diffuse hyperintense signals on T2-weighted imaging (T2WI) in the cerebral white matter (white matter hyperintense signal [WMHS]). Currently, the pathological basis of this phenomenon remains unclear, and few studies have systematically investigated its impact on fetal and postnatal brain development. We aimed to compare the differences in apparent diffusion coefficient (ADC) values across distinct brain regions between fetuses with WMHS and age-matched control fetuses, to explore the potential etiology of WMHS by combining these findings with morphological changes in cerebral veins, and to conduct a comprehensive evaluation combined with clinical follow-up.

**Method:**

We retrospectively analyzed fetal imaging data from MRI examinations performed at our hospital between January 2014 and May 2024. A total of 87 late-term fetuses (gestational age [GA]: 29–40 weeks) were identified with diffuse hyperintense signals on T2WI in the cerebral parenchyma; age-matched normal fetuses with the same sample size were enrolled as the control group. We compared differences in ADC values across distinct brain regions among fetuses with WMHS and simultaneously analyzed morphological differences in the deep cerebral veins between the two groups, combined with postnatal clinical follow-up, to explore the impact of WMHS on brain development and its clinical significance in late-term fetuses.

**Results:**

(1) ADC values in the majority of brain regions of fetuses in the WMHS group were higher than those in the control group, with the greatest difference observed in the F2 region. (2) Compared with the control group, fetuses in the WMHS group exhibited significantly increased lumen areas of the vein of Galen (VOG) and straight sinus (SS), with a *p*-value of < 0.05 considered statistically significant. (3) Successful follow-up was achieved in 45 control infants and 36 WMHS cases. All control fetuses showed normal neurodevelopmental outcomes, while 33 infants in the WMHS group had normal and 3 had adverse outcomes, including cerebral palsy, developmental delay, and autism.

**Conclusion:**

In late-term fetuses with WMHS, elevated ADC values in specific brain regions and concurrent deep cerebral venous dilation indicate underlying intracranial abnormalities. In addition, our follow-up results indicate that these combined changes may be associated with adverse neurodevelopmental outcomes in some infants.

## 1 Introduction

Clinically, we have observed that some late-term fetuses with normal cerebral morphology exhibit diffuse hyperintense signals on T2-weighted imaging (T2WI) in the cerebral parenchyma on prenatal MRI. This phenomenon was defined as a white matter hyperintense signal (WMHS) by Katorza et al. (1, 2), who hypothesized that cerebral edema is one of the underlying causes. Diffusion-weighted imaging (DWI) is a commonly used sequence for assessing restricted water molecule diffusion. The apparent diffusion coefficient (ADC) value can be quantitatively measured. When brain parenchymal injury occurs, it may affect water molecule diffusion in the fetal brain tissue, resulting in an increase or decrease in the ADC value. The intracranial arteriovenous system plays a crucial role in ensuring blood circulation and maintaining the stability of the fetal cerebral microenvironment; however, whether corresponding changes occur in fetal cerebral hemodynamics during this period remains unclear. The vein of Galen (VOG) and straight sinus (SS) are the main pathways for deep cerebral venous blood drainage (3, 4). After the occurrence of cerebral injury, a series of corresponding changes, such as perfusion alterations and abnormal blood flow, may occur, which could potentially affect fetal brain development and postnatal quality of life (5, 6).

In this study, we aimed to explore the diagnostic significance of WMHS by measuring ADC values in different brain regions of fetuses with WMHS, combined with cerebral venous parameter data and postnatal clinical follow-up of these fetuses.

## 2 Materials and methods

### 2.1 Baseline data

This was a retrospective study approved by the Institutional Ethics Committee of our hospital. All pregnant women participating in the study provided written informed consent prior to the examination for using their clinical data for research purposes. All MRI images were retrieved from our fetal MRI database, including those of fetuses who underwent MRI between January 2014 and May 2024, with a gestational age ranging from 29 to 40 weeks. The fetuses were divided into two groups: the WMHS group and the healthy control group. All fetal brain MR images were selected and verified by two radiologists with 10 years of experience in fetal brain MR diagnosis. The majority of fetal MRI scans were performed due to suspected intracranial abnormalities detected by prenatal ultrasonography, including ventriculomegaly, cisterna magna enlargement, and cavum septi pellucidi dilation. A small proportion of cases underwent fetal MRI for voluntary prenatal health screening at the request of the families. In the control group, only fetuses with no abnormal findings on both fetal MRI and subsequent prenatal ultrasound follow-up were included to ensure the absence of intracranial or other relevant abnormalities. Diffuse hyperintense signals on T2WI in the cerebral white matter of fetuses in the WMHS group were confirmed repeatedly by the radiologists.

### 2.2 Inclusion criteria for the WMHS group

The inclusion criteria for the WMHS group include the following:

Singleton pregnancy with a definite gestational age.
Normal development of fetal cerebral parenchymal structure accompanied by the WMHS.
Maternal age > 18 years.
Gestational age of > 29 weeks and < 40 weeks.
No family history of genetic diseases.

In addition, during the selection of the control group, controls were strictly matched with WMHS cases based on key factors, including age, GA (week), pre-pregnancy body mass index (BMI), smoking history, drinking history, number of previous pregnancies, number of deliveries, pre-pregnancy chronic diseases, and family genetic history. Pregnant women with high-risk factors during pregnancy, such as pregnancy-induced hypertension and gestational diabetes, were excluded. When multiple eligible candidates were available, control participants with the closest similarity in these variables were selected to ensure optimal matching, thereby minimizing potential bias in the results.

#### 2.2.1 Inclusion criteria for the control group

Fetal MRI revealed typical white matter hyperintense signals (WMHS) consistent with the study definition, as diagnosed by two independent experienced radiologists.

Maternal age > 18 years.

Gestational age at MRI between 29 weeks and 40 weeks.

#### 2.2.2 Exclusion criteria for the WMHS group

The exclusion criteria for the WMHS group include the following:

Fetuses with additional intracranial structural or hemorrhagic abnormalities, including germinal matrix hemorrhage–intraventricular hemorrhage (GMH-IVH), punctate white matter lesions, ventriculomegaly, cerebral malformations, cortical malformations, or other parenchymal lesions.
Fetuses with severe congenital anomalies or syndromic disorders outside the central nervous system that may confound neurodevelopmental outcomes.
Cases with incomplete imaging data.

### 2.3 MR imaging protocol

All fetal head and neck MRI scans were performed using a GE Signa 1.5 T MR scanner. Images in the axial, coronal, and sagittal planes were obtained using the balanced steady-state free precession [fast imaging employing steady-state acquisition (FIESTA)] sequence and single-shot fast spin-echo (SSFSE) sequence]. Axial images were obtained using the fast inversion recovery motion (FIRM) suppression sequence and diffusion-weighted imaging (DWI) sequence. The scanning order and parameters are detailed as follows: FIESTA: repetition time (TR) = 4.78 ms, echo time (TE) = 2.16 ms, field of view (FOV) = 38 cm × 38 cm, matrix = 256 × 224, slice thickness = 5 mm; SSFSE: TR = 2000 ms, TE = 159.71 ms, FOV = 38 cm × 38 cm, matrix = 256 × 256, slice thickness = 5 mm; and FIRM: TR = 150 ms, TE = 4.2 ms, FOV = 38 cm × 38 cm, matrix = 288 × 180, slice thickness = 3 mm; DWI: TR = 3,274 ms, TE = 75 ms, b-values = 0 and 700 s/mm^2^, and FOV = 38 cm × 38 cm.

### 2.4 Image analysis

**Figure 1:**
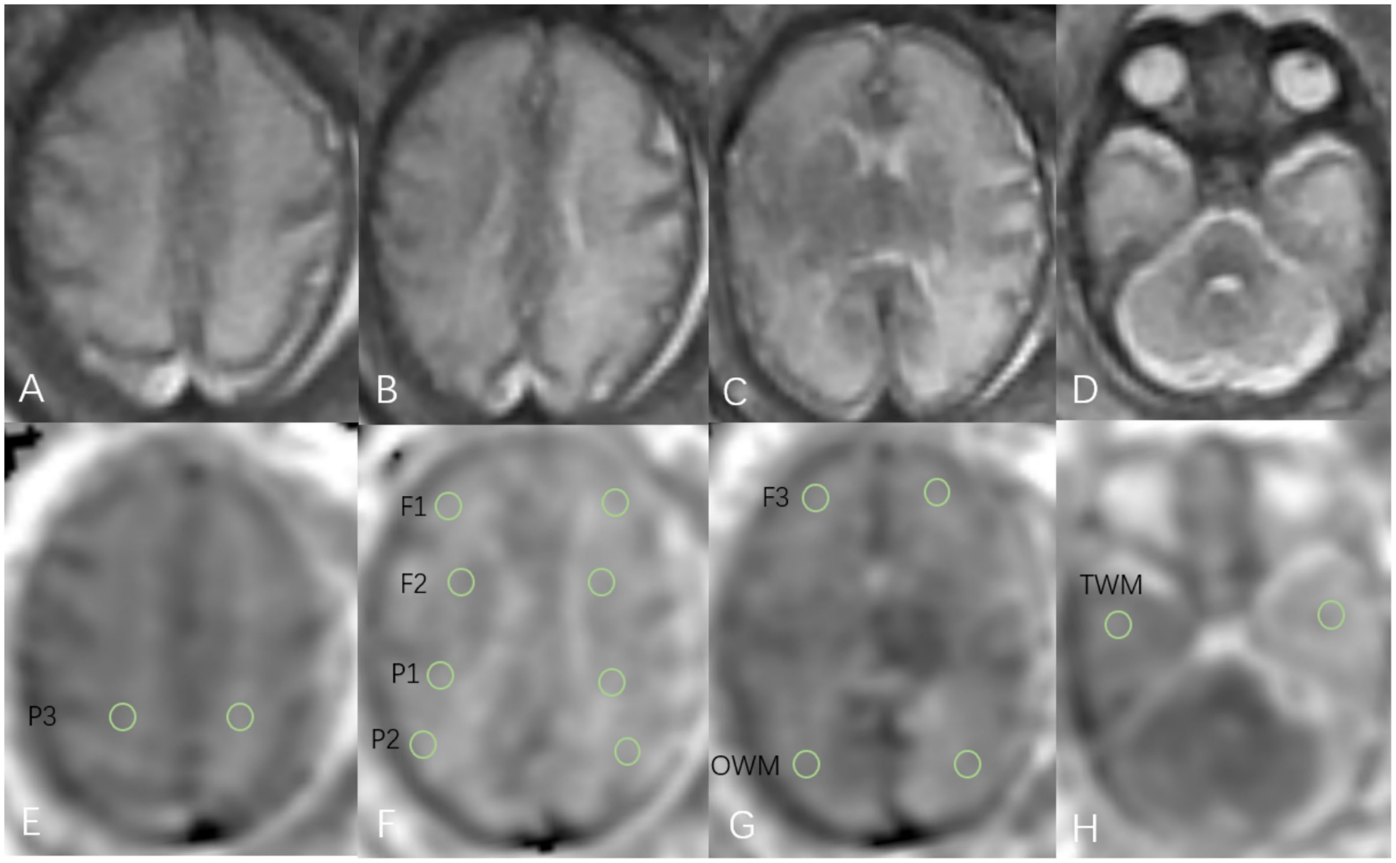
Schematic diagram of axial SSFSE sequence **(A–D)** and ADC **(E–H)** measurement in a 32-week fetus with WMHS. On the SSFSE sequence, diffuse hyperintense T2WI signals in the cerebral white matter of both cerebral hemispheres were observed. The ADC measurement diagram illustrates the ROIs delineated in the corresponding brain areas.

Diffusion-weighted imaging (DWI) data were transferred to a GE workstation for analysis, and apparent diffusion coefficient (ADC) measurements were recorded (Figures 1A–H). Regions of interest (ROIs) were manually delineated with an area ranging from 30 to 50 mm^2^. A total of 16 subcortical white matter ROIs were selected across four planes (centrum semiovale plane, lateral ventricular body plane, basal ganglia–thalamus plane, and cerebellar plane), and the mean ADC value of each ROI was calculated. Specifically, there were three pairs of frontal lobe white matter ROIs: F1 and F2 (Figure 1F) are located in the anterior corona radiata of the lateral ventricular body plane and F3 (Figure 1G) is located at the anterior end of the basal ganglia plane; three pairs of parietal lobe white matter ROIs: P1 and P2 (Figure 1F) are located in the posterior corona radiata of the lateral ventricular body plane, and P3 (Figure 1E) is located in the centrum semiovale plane; and one pair of occipital lobe white matter (OWM) ROIs (Figure 1G) in the basal ganglia plane and one pair of temporal lobe white matter (TWM) ROIs (Figure 1H) in the brainstem-cerebellar plane were included (5).

To ensure the reproducibility of measurements, all ADC values were remeasured by the same radiologist 1 week later. If discrepancies existed between the two measurements, a third measurement was performed, and the average value was taken.

**Figure 2:**
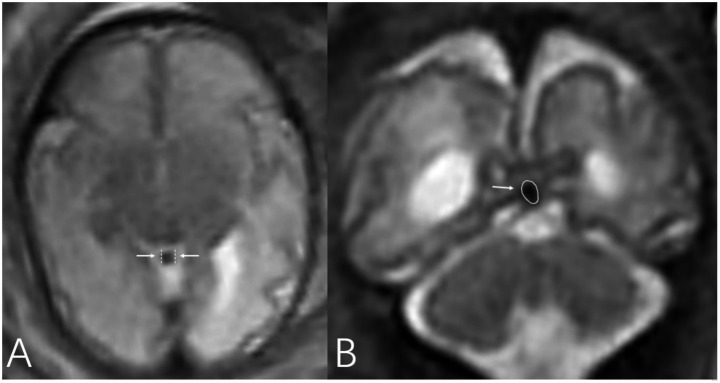
Axial and coronal SSFSE sequences of a 35-week fetus with WMHS. Diffuse dilation of the VOG **(A)** and SS **(B)** is evident on the images. Measurement methods: On the axial plane, the distance between the two arrows indicates the diameter of the fetal VOG, and its cross-sectional area was calculated using the formula: area = πr^2^. On the coronal plane, the cross-section of the fetal SS is displayed, and its cross-sectional area was measured directly.

The single-shot fast spin-echo (SS-FSE) sequence, which provides excellent venous visualization, was used to measure the diameter of the vein of Galen (VOG) and the cross-sectional area of the straight sinus (SS). The VOG is located below the splenium of the corpus callosum, extends posterosuperiorly to form a thicker and more prominent vein, and runs inferiorly and posteriorly along the tentorium cerebelli to converge with the inferior sagittal sinus, forming the SS. The SS is a venous channel between the dural layers, which is mostly triangular during the mid-to-late gestational period (6). Therefore, the diameter of the VOG (Figure 2A) was measured on the axial plane at the proximal segment near its confluence with the SS; the cross-sectional area of the SS (Figure 2B) was measured on the oblique coronal plane. Since the cross-section of the VOG is circular, its area was converted from the measured diameter (R) using the formula πr^2^ to facilitate the comparison of dilation degrees between the VOG and SS. All aforementioned measurements were performed by the same radiologist who measured the ADC values, following the same standards. Clinical information of the patients was masked during all imaging measurements to ensure blinding.

### 2.5 Statistical methods

Data were analyzed and processed using SPSS Statistics 26 software. Paired t-tests revealed no significant differences in ADC values between symmetric brain regions on the left and right sides; therefore, ADC values from symmetric anatomical locations in bilateral brain regions were averaged. First, the normality of distribution was tested for ADC values in each brain region, VOG diameter, and SS cross-sectional area. Quantitative data with a normal distribution were expressed as mean ± standard deviation ( $\bar{x}$ ±s), and intergroup comparisons were performed using independent-samples t-tests. Quantitative data with a non-normal distribution were expressed as median (quartile range) [M(Q_L_, Q_U_)], and intergroup comparisons were conducted using the Mann–Whitney U test. A *p*-value of < 0.05 was considered statistically significant.

### 2.6 Follow-up

Clinical follow-up was performed through the pediatric developmental clinic or telephone follow-up to assess childhood scale scores after birth. For children aged ≤6 years, neurodevelopmental assessment was conducted using the Gesell scale, including five domains: adaptability, gross motor, fine motor, language, and personal-social skills. Neurodevelopmental level was evaluated using the development quotient (DQ) score (development quotient = mental age / chronological age). A DQ of > 85 indicated normal development, 85 ≥ DQ ≥ 76 indicated a borderline state, 75 ≥ DQ ≥ 55 indicated mild developmental delay, 54 > DQ ≥ 40 indicated moderate developmental delay, and 39 > DQ ≥ 29 indicated severe developmental delay.

## 3 Results

### 3.1 General demographic data

We retrospectively identified 87 pregnant women in the WMHS group and 87 in the control group who met the inclusion criteria of the study, all of which were verified by physicians to fulfill the eligibility requirements. In the WMHS group (*n* = 87), the mean maternal age was 29.75 ± 2.56 years (range: 23–43 years), and the mean gestational age was 33.56 ± 2.43 weeks (range: 29–38 weeks). In the control group (*n* = 87), the mean maternal age was 30.36 ± 3.07 years (range: 24–40 years), and the mean gestational age was 33.25 ± 2.01 weeks (range: 29–38 weeks). No significant differences were observed between the two groups (*p* > 0.05) (Table 1).

**Table 1:** Comparison of general demographic data.

Characteristics	Group		*p*
	Control (*n* = 87)	WMHS (*n* = 87)	
Age (year)	30.36 ± 3.07	29.75 ± 2.56	0.138
GA (week)	33.25 ± 2.01	33.56 ± 2.43	0.165
Pre-pregnancy BMI	120.16 ± 1.07	20.46 ± 1.08	0.067
Number of previous pregnancies	0.47 ± 0.61	0.49 ± 0.63	0.850
Number of deliveries	0.32 ± 0.49	0.34 ± 0.52	0.760
Smoking history	12	15	0.520
Drinking history	21	28	0.220
Pre-pregnancy chronic diseases	11	16	0.290
Family genetic history	0	2	0.246

### 3.2 ADC values in various brain regions

Independent-samples t-tests and Mann–Whitney U tests revealed that, compared with the control group, fetuses in the WMHS group exhibited significantly higher ADC values in the F1, F2, F3, P1, P2, P3, TWM, and OWM regions (all *p* < 0.001). The greatest differences were observed in the F2, P1, and F3 regions, with difference rates of 11.89 ± 6.96%, 10.93 ± 5.65%, and 10.68 ± 7.23%, respectively. The smallest difference rate was noted in the TWM region (3.91 ± 1.59%).

**Figure 3:**
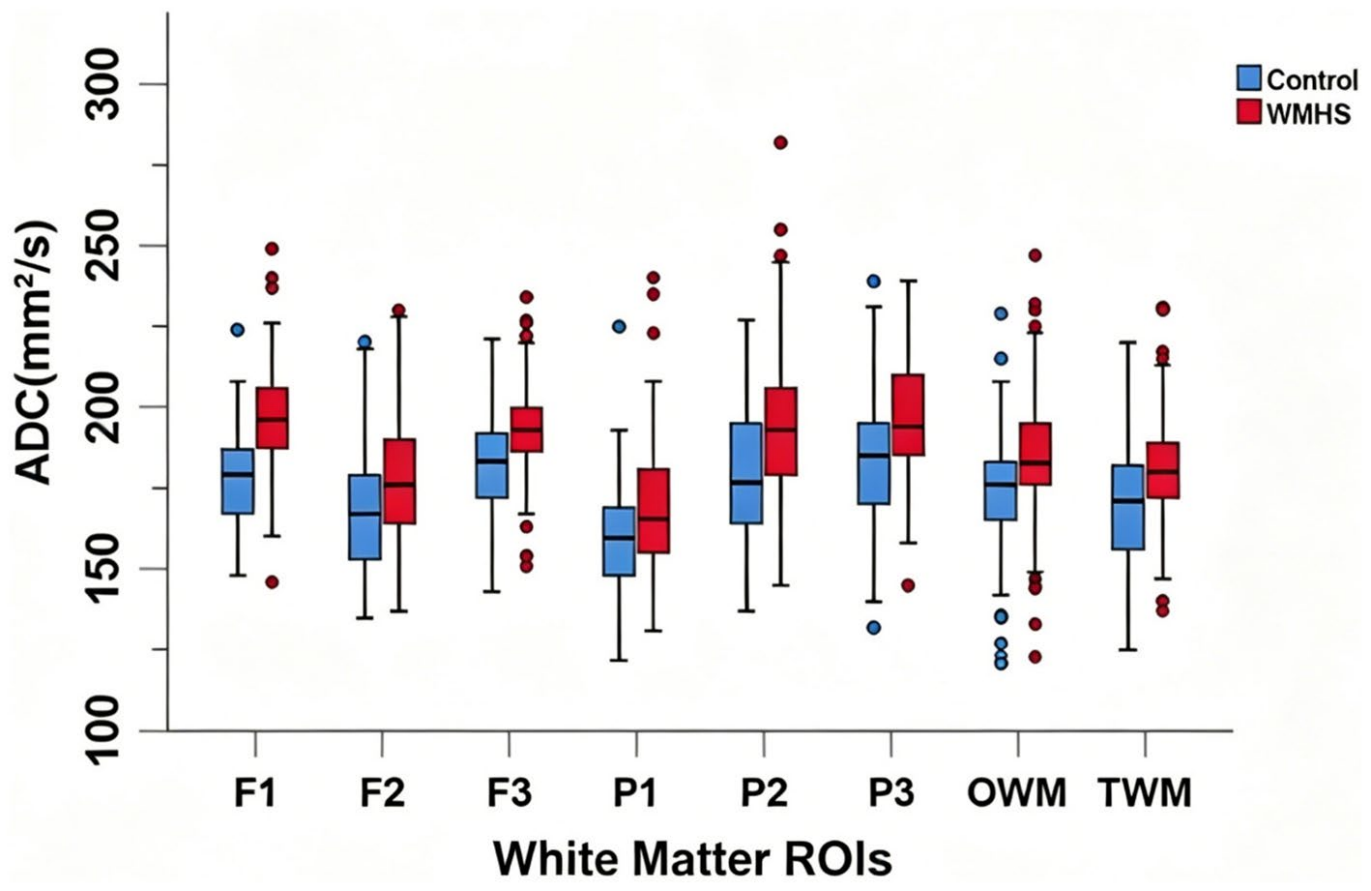
Comparison of ADC values in white matter ROIs between the control group (*n* = 87) and the WMHS group (*n* = 87).

The four pairs of ROIs adjacent to the lateral ventricles showed relatively high difference rates (mean: 9.89 ± 6.65%), which were higher than those of other ROIs (6.75 ± 3.93%) (Table 2) (see Figure 3).

**Table 2:** Detailed ADC values of white matter ROIs in the control group (*n* = 87) and the WMHS group (*n* = 87).

ROI	Control [ $\bar{x}$ ±s]	WMHS[ $\bar{x}$ ±s]	Discrepancy rate	*p*
F1 (s/mm^2^)	176.69 ± 11.10	191.63 ± 8.95	8.46 ± 8.90%	<0.001^*^
F2 (s/mm^2^)	168.09 ± 8.80	188.06 ± 6.02	11.89 ± 6.96%	<0.001^*^
F3 (s/mm^2^)	174.48 ± 10.87	193.12 ± 10.75	10.68 ± 7.23%	<0.001^*^
P1 (s/mm^2^)	171.29 ± 8.88	190.02 ± 8.49	10.93 ± 5.65%	<0.001^*^
P2 (s/mm^2^)	176.52 ± 7.86	191.14 ± 7.13 ±22.73	8.28 ± 5.12%	<0.001^*^
P3 (s/mm^2^)	182.99 ± 19.96	195.98 ± 17.61	7.10 ± 3.21%	<0.001^*^
OWM (s/mm^2^)	173.29 ± 18.09	184.20 ± 20.14	5.29 ± 3.67%	<0.001^*^
TWM (s/mm^2^)	174.77 ± 1,077	181.61 ± 17.54	3.91 ± 1.59%	<0.001^*^

^*^Indicates a statistically significant difference.

### 3.3 Fetal venous morphology

The Mann–Whitney U tests demonstrated that the cross-sectional areas of the VOG and SS were significantly larger in the WMHS group than those in the control group (all *p* < 0.0001). After unit conversion to area for the comparison of dilation degrees, the VOG and SS in the WMHS group exhibited dilation rates of 26.44 ± 11.87% and 29.24 ± 13.62%, respectively, with the SS showing a higher dilation degree than the VOG.

**Figure 4:**
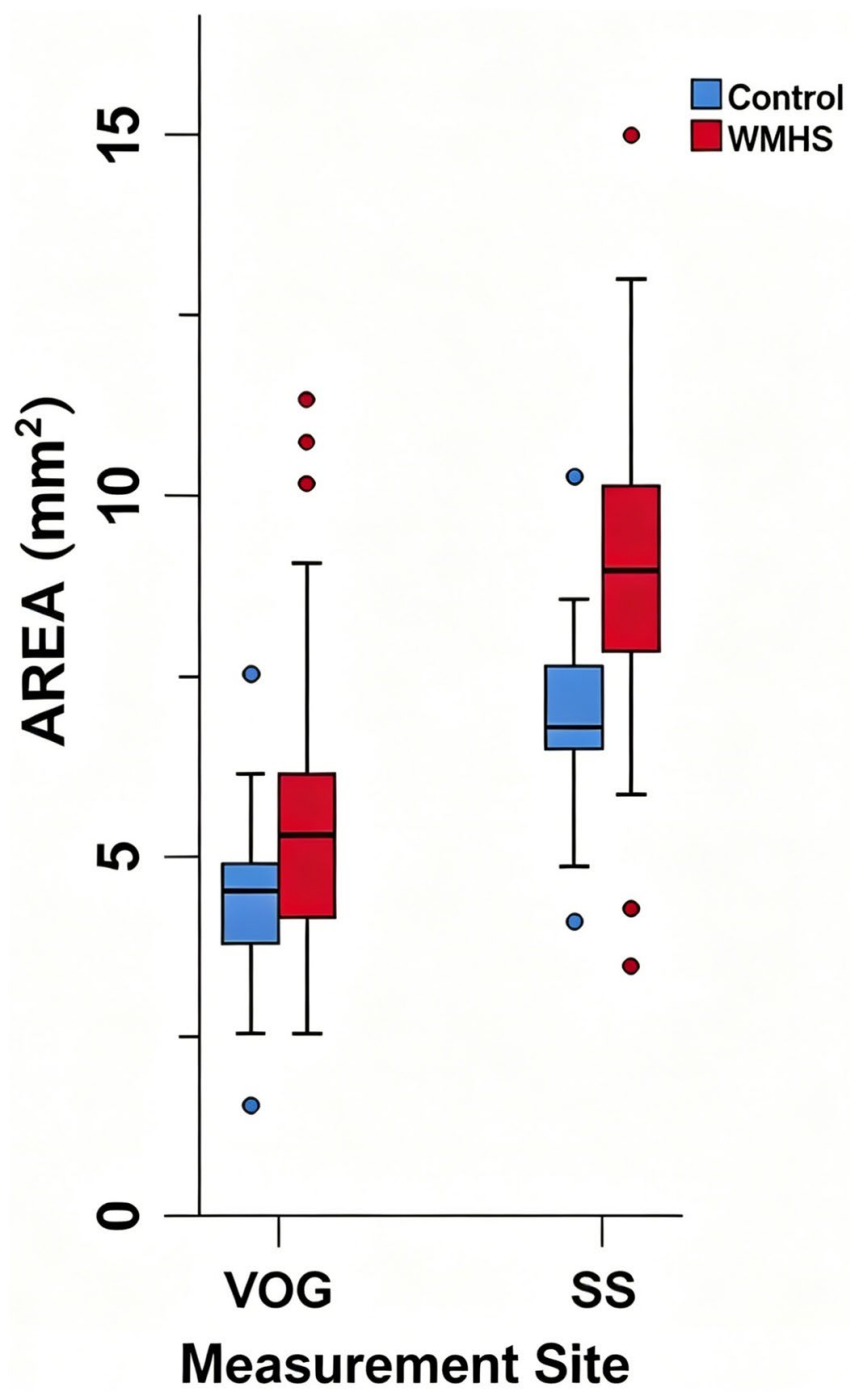
Comparison of cross-sectional areas of the vein of Galen and straight sinus between the control group (*n* = 87) and the WMHS group (*n* = 87).

When comparing the control group with the WMHS group, the dilation rates of the VOG and SS were 6.46 ± 2.19% and 15.00 ± 2.19%, respectively; the SS also showed a higher dilation degree than the VOG, but no statistically significant differences were observed between the two groups (*p* > 0.05) (Table 3) (see Figure 4).

**Table 3:** Comparison of cross-sectional areas of the vein of galen and straight sinus between the control group and WMHS group.

ROI	Control ( $\bar{x}$ ±s)	WMHS ( $\bar{x}$ ±s)	Discrepancy rate	*p*
VOG (s/mm^2^)	4.35 ± 0.94	5.50 ± 2.02	26.44 ± 11.87%	<0.0001
SS (s/mm^2^)	7.01 ± 0.91	9.06 ± 1.89	29.24 ± 13.62%	<0.0001

### 3.4 Follow-up

Clinical follow-up was conducted in 87 fetuses in the control group and 87 in the WMHS group, with 45 and 36 cases successfully followed up, respectively.

In the control group (age range: 0–6 years), there were 43 term deliveries, 2 preterm deliveries, 31 vaginal deliveries, and 14 cesarean deliveries. All infants had normal Apgar scores at birth, and normal neurodevelopmental outcomes were demonstrated during follow-up.

In the WMHS group (age range: 0–5 years), there were 25 term deliveries, 11 preterm deliveries, 22 vaginal deliveries, and 14 cesarean deliveries. Among the 36 live-born infants, 28 had normal Apgar scores and 6 had abnormal Apgar scores at birth.

According to the Gesell developmental assessment, 33 infants in the WMHS group exhibited normal development (developmental quotient ≥ 85). Of the 6 infants with abnormal Apgar scores, 4 showed transient abnormalities (low scores at 1 or 5 min that normalized at 10 min) and exhibited normal development on evaluation.

One infant with persistently low Apgar scores (3–4-5) was delivered at 35 weeks of gestation (preterm and low birth weight) by cesarean section with an uneventful labor course. At the follow-up age of 66 months, the infant had been diagnosed with cerebral palsy at 28 months. Physical examination revealed increased muscle tone in both lower extremities, tiptoe walking, knee internal rotation, and a mild spastic gait. Neurological examination showed hyperactive knee tendon reflexes and weakly positive bilateral Babinski signs. The Gesell assessment indicated severe developmental delay in gross and fine motor function.

One infant with persistently low Apgar scores (5–6-6) was delivered at term with normal birth weight by cesarean section with an uneventful labor course. At the follow-up age of 8.5 months, the Gesell assessment showed borderline developmental delay in gross motor function and mild developmental delay in fine motor function.

Another infant in the WMHS group with normal Apgar scores at birth was delivered by cesarean section at term, with normal birth weight and an uncomplicated delivery process. At the follow-up age of 29 months, the infant had been diagnosed with autism at 24 months. Physical examination demonstrated poor interaction, decreased social interest, repetitive stereotyped behaviors, abnormal sensory perception (sensitivity to specific sounds with ear covering or crying), and impaired self-care ability. The Gesell assessment revealed moderate developmental delay in language and social function (Table 4).

**Table 4:** Gesell developmental assessment scale.

Fetal No.	Tested developmental domain	Developmental age (DA)	Developmental quotient (DQ)	Evaluation (developmental delay)
1	Adaptive behavior	44.9	68	Mild
	Gross motor skills	25.1	38	Severe
	Fine motor skills	20.5	31	Severe
	Language	35.0	53	Moderate
	Personal-social skills	33.7	51	Moderate
2	Adaptive behavior	8.5	100	Normal
	Gross motor skills	6.8	80	Borderline
	Fine motor skills	6.2	73	Mild
	Language	7.3	86	Normal
	Personal-social skills	8.1	95	Normal
3	Adaptive behavior	16.5	57	Moderate
	Gross motor skills	22.0	76	Borderline
	Fine motor skills	197	67	Mild
	Language	8.1	28	Severe
	Personal-social skills	10.4	36	Severe

## 4 Discussion

WMHS is a prenatal MRI imaging feature characterized by diffuse T2WI hyperintensity in the fetal cerebral white matter with normal brain morphology, first defined by Katorza et al. (1), and its pathological basis remains unclear. Katorza et al. (1) proposed that cerebral edema caused by increased extracellular fluid in white matter may be an important cause of WMHS and also reported that approximately half of WMHS cases were associated with congenital CMV infection, indicating its multi-etiological characteristics. The immature fetal blood–brain barrier (BBB) is prone to disruption and increased permeability induced by various insults, leading to cytotoxic and vasogenic biphasic edema (7, 8). DWI is sensitive to cerebral edema: cytotoxic edema initially reduces ADC values due to restricted water diffusion, whereas chronic-phase vasogenic edema increases ADC values by expanding the extracellular space (2). ADC changes reflect the proportion of edema and cellular components in brain tissue (9, 10).

An elevated ADC value is a classic and well-recognized imaging hallmark of vasogenic edema in fetal brain imaging, which is essentially different from the decreased ADC value caused by cytotoxic edema with restricted water molecule diffusion (7, 10, 11). The most significant ADC elevation in the periventricular region in our study is closely related to its anatomical characteristics: the germinal matrix in this region has an immature blood–brain barrier and a fragile capillary network, which is prone to barrier disruption and extracellular fluid accumulation—the core pathological feature of vasogenic edema (12, 13). The normal cerebral parenchymal structure of fetuses in the WMHS group further rules out the interference of structural lesions such as malformation and infarction on ADC values.

Fetal WMHS may be caused by various factors, including hypoxia, cytomegalovirus (CMV) infection, and various leukodystrophies (1, 2). Hypoxia can lead to BBB disruption and increased permeability, thereby causing secondary cytotoxic and vasogenic edema, resulting in focal or diffuse WMHS (1, 2, 11, 12, 14, 15). WMHS caused by CMV infection typically originates in the temporal lobe, characterized by prominent T2 hyperintensity in the temporal lobe, often accompanied by temporal horn dilation and temporal lobe volume reduction; the hyperintensity gradually spreads to the entire brain parenchyma (1, 16). Leukodystrophies may also cause fetal WMHS, including Alexander disease, Krabbe disease, and Canavan disease (17–19). The majority of these conditions exhibit specific imaging distribution characteristics and family histories of inheritance; therefore, genetic testing should be considered for fetuses with WMHS prenatally.

We found a close association between WMHS and intracranial venous dilation. Fetal ischemia–hypoxia is a well-documented cause of deep cerebral venous morphological changes: Doneda et al. (12) confirmed that fetal ischemia–hypoxia can induce cerebral venous hypertension, leading to the dilation of the vein of Galen and straight sinus, and subsequent blood–brain barrier disruption and vasogenic edema, which is consistent with the concurrent changes of venous dilation and elevated ADC values in the WMHS group in our study. Cerebral venous dilation is a typical hemodynamic response to ischemia–hypoxia, reflecting the decompensation of cerebral blood circulation after the failure of arterial blood flow compensation (20–22), and the higher dilation degree of the straight sinus is due to its physiological characteristics as the confluence of multiple deep cerebral veins, which makes it more susceptible to the influence of ischemia–hypoxia-induced hemodynamic changes (20). Significant dilation of the deep cerebral veins was observed in the fetuses, with the measured values of the vein of Galen (VOG) and straight sinus (SS) being 26.56% and 28.82% higher than the normal values in the control group, respectively. There are few morphological studies on cerebral veins in fetuses with craniocerebral diseases. Abnormalities in cerebral veins can be observed in some conditions. One report described a fetus with severe heart failure involving the cerebral veins, who showed obvious cerebral edema on MRI, and one of them had dilation of the VOG and SS. Due to intrauterine death occurring in the fetus a few days after the MRI examination, the authors suggested that venous hypertension during this process induced medullary venous swelling and venous infarction, which may indicate a poor prognosis (12). Cheema et al. (20) observed fetuses with growth restriction using ultrasound and found frequent pulsations and increased blood flow velocity in the VOG, SS, and transverse sinus, with the VOG showing the most obvious changes. They proposed that the blood flow velocity in the VOG can reflect changes in fetal central venous pressure, cerebral blood flow, and cerebrovascular resistance (19). In our study, MRI also showed dilation of the VOG and SS in fetuses with WMHS, with the SS exhibiting a higher dilation degree than the VOG. A possible reason is that the SS is formed by the confluence of multiple veins, including the VOG, inferior sagittal sinus, and basal vein, and collects blood flow from the deep veins of the entire brain, making it more susceptible to influence (6). We hypothesize that the association between SS dilation and WMHS may be more significant than that between VOG dilation and WMHS; however, this requires confirmation with more case data (22–24).

Our postnatal follow-up data further suggest the clinical relevance of fetal WMHS-related imaging abnormalities. All successfully followed-up control infants achieved normal neurodevelopmental outcomes, whereas 8.33% of WMHS cases presented adverse neurodevelopmental outcomes, including cerebral palsy, developmental delay, and autism. Notably, two of these abnormal cases were accompanied by persistently low Apgar scores, which may indicate a potential association between perinatal hypoxic events, fetal intracranial venous dilation, elevated ADC values, and postnatal neurological impairment. The third case with normal Apgar scores but autistic features suggests that WMHS may represent an independent prenatal imaging marker for subtle neurodevelopmental disorders that are not associated with acute perinatal asphyxia. It should be emphasized that adverse neurodevelopmental outcomes are multifactorial and cannot be attributed solely to fetal WMHS; multiple well-documented perinatal factors, including perinatal asphyxia, prematurity, low birth weight, and other perinatal complications, may also contribute to postnatal neurological impairment. These preliminary findings suggest that fetal MRI-detected WMHS, combined with elevated regional ADC values and deep cerebral venous dilation, may not represent an entirely benign imaging finding but may be associated with an increased risk of long-term neurodevelopmental sequelae. The etiology and independent predictive value of WMHS require further investigation with larger samples and adjusted confounders.

Fetuses with WMHS exhibit elevated ADC values in the corresponding hyperintense brain regions and dilation of the deep cerebral veins. Previous MRI studies have rarely focused on the intracranial venous changes associated with fetal WMHS. In this study, we linked fetal WMHS to changes in the cerebral venous system and conducted a quantitative analysis with a large sample size, which will facilitate future research and treatment of fetal WMHS. However, this study still has certain limitations: (1) The sample data only include fetuses in the late gestational period, and fetuses in the mid-gestational period were not included due to the limited sample size; (2) as a retrospective study, our MRI examination protocol did not include quantitative susceptibility mapping (QSM) sequences related to oxygenation; and (3) a lack of long-term, large-sample follow-up results was observed. Nevertheless, we will continue to focus on this area to address these limitations.

## 5 Conclusion

In late-term fetuses with WMHS, elevated ADC values in specific brain regions and concurrent deep cerebral venous dilation indicate underlying intracranial abnormalities. In addition, our follow-up results indicate that these combined changes may be associated with adverse neurodevelopmental outcomes in some infants.

## Data Availability

The original contributions presented in the study are included in the article/supplementary material, further inquiries can be directed to the corresponding author.
